# GPVI surface expression and signalling pathway activation are increased in platelets from obese patients: Elucidating potential anti-atherothrombotic targets in obesity

**DOI:** 10.1016/j.atherosclerosis.2018.12.023

**Published:** 2019-02

**Authors:** María N. Barrachina, Aurelio M. Sueiro, Irene Izquierdo, Lidia Hermida-Nogueira, Esteban Guitián, Felipe F. Casanueva, Richard W. Farndale, Masaaki Moroi, Stephanie M. Jung, María Pardo, Ángel García

**Affiliations:** a Platelet Proteomics Group, Center for Research in Molecular Medicine and Chronic Diseases (CIMUS), Universidade Santiago de Compostela, Santiago de Compostela, Spain; b Instituto de Investigación Sanitaria de Santiago (IDIS), Santiago de Compostela, Spain; c Grupo de Endocrinología Molecular y Celular – Instituto de Investigación Sanitaria de Santiago (IDIS)/ Servicio de Endocrinología, Xerencia de Xestión Integrada de Santiago (XXS), Santiago de Compostela, Spain; d Mass Spectrometry and Proteomic Unit, Rede de Infraestructuras de Apoio á Investigación e ao Desenvolvemento Tecnolóxico, Universidade de Santiago de Compostela, Santiago de Compostela, Spain; e Department of Biochemistry, University of Cambridge, Cambridge, CB2 1QW, United Kingdom; f Grupo Obesidómica - Instituto de Investigación Sanitaria de Santiago (IDIS), Santiago de Compostela, Spain

**Keywords:** Platelets, Obesity, Proteomics, GPVI signalling, SFK-Mediated signalling pathways, Drug targets

## Abstract

**Background and aims:**

Platelets play a fundamental role in the increased atherothrombotic risk related to central obesity since they show hyperactivation and lower sensitivity to antiplatelet therapy in obese patients. The main goal of this study was to identify platelet biomarkers related to the risk of atherothrombosis in obese patients, confirm platelet activation levels in these patients, and identify altered activation pathways.

**Methods:**

Platelets were obtained from cohorts of obese patients and age- and sex-matched lean controls. Biochemical and proteome analyses were done by two-dimensional differential in-gel electrophoresis (2D-DIGE), mass spectrometry, and immunoblotting. Functional and mechanistic studies were conducted with aggregation assays and flow cytometry.

**Results:**

We confirmed an up-regulation of αIIb and fibrinogen isoforms in platelets from obese patients. A complementary platelet aggregation approach showed platelets from obese patients are hyper-reactive in response to collagen and collagen-related peptide (CRP), revealing the collagen receptor Glycoprotein VI (GPVI) signalling as one of the altered pathways. We also found the active form of Src (pTyr418) is up-regulated in platelets from obese individuals, which links proteomics to aggregation data. Moreover, we showed that CRP-activated platelets present higher levels of tyrosine phosphorylated PLCγ2 in obese patients, confirming alterations in GPVI signalling. In line with the above, flow cytometry studies show higher surface expression levels of total GPVI and GPVI-dimer in obese platelets, both correlating with BMI.

**Conclusions:**

Our results suggest a higher activation state of SFKs-mediated signalling pathways in platelets from obese patients, with a primary involvement of GPVI signalling.

## Highlights

• Higher expression of the active form of Src (pTyr418) in obese patients platelets.
• Increased aggregation levels of obese platelets in response to GPVI activation.
• GPVI-activated platelets show higher levels of tyr-phosphorylated PLCγ2 in obesity.
• Higher surface expression levels of GPVI in obese platelets, correlating with BMI.

## Graphical abstract

**Figure:**
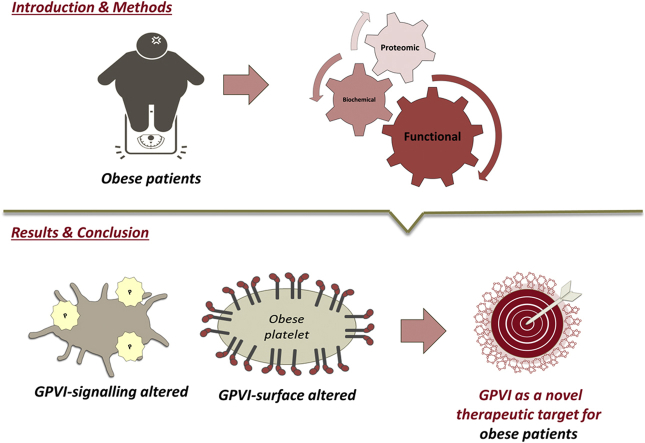

## 1 Introduction

Obesity represents one of the biggest health problems in industrialized countries. Indeed, the World Health Organization estimates that more than 1 billion adults worldwide are overweight, 300 million of whom are clinically obese (defined as having a body mass index (BMI) ≥30 kg/m^2^) [1].

Central obesity corresponds to an excess of intra-abdominal adipose tissue [2]. This accumulation leads to the intrinsic dysfunction of adipose tissue, together with insulin resistance, endothelial dysfunction, systemic inflammatory state and oxidative stress. It is well known that those obesity-associated alterations constitute a relevant risk factor for suffering cardiovascular diseases (CVD), especially, the development of atherothrombosis [3]. In fact, the link between obesity and CVD has been repeatedly evidenced and associated with the chronic exposure to a pro-inflammatory and pro-thrombotic state in obese subjects [4].

Obese individuals tend to have platelet abnormalities in mean platelet volume and higher rates of ischemic events [5,6]. Increased platelet activation has been found in obese women free of cardiovascular risk factors [7]. Furthermore, recent studies focused the attention on the pathogenetic role of platelet hyperactivation and reduced sensitivity to antiaggregating therapy in obese patients [4,8].

The main goal of the present study was to identify platelet biomarkers related to the risk atherothrombosis in obese patients without CVD. Initial proteomic results were validated by immunoblotting on larger cohorts of patients. Moreover, complementary flow cytometry (FACs) and aggregation studies were undertaken to confirm platelet activation levels on these patients, and identify the most altered activation pathways.

## 2 Materials and methods

### 2.1 Patients

The study was approved by the Galician Investigation Ethics Committee and followed the Declaration of Helsinki principles. All participants provided written informed consent.

Thirty-four patients referred to the Endocrinology Unit at the Santiago de Compostela University Hospital, Spain, participated in the study. These patients had a BMI higher than 40, indicating severe “healthy” obesity, and were referred to the hospital by the primary care physician so they could be treated by an endocrinologist. Exclusion criteria were coagulation disorders, platelet-associated disorders, chronic antiplatelet drugs and other chronic drug therapy (except for drugs required to treat pre-existing clinical factors that do not affect platelet reactivity). Only 1 patient was diabetic, with no relevant impact on the results; this patient was included in the pooled samples but not in any individual validation approach. The lean control group consisted of healthy normal weight volunteers recruited at the Santiago de Compostela University Health Service. This group was age- and gender-matched with the obese group and individuals had a BMI between 18 and 26.

Additionally, another group of 11 healthy volunteers with BMI between 26 and 35 (overweight and grade 1 obese individuals) was included to investigate the expression levels of GPVI on the platelet surface. This group, age-matched with the other groups, was also recruited at the Santiago de Compostela University Health Service.

The initial proteomic screening included 10 obese patients and 10 lean matched-controls (Supplementary Table 1). Immunoblotting validations and functional studies included 34 obese patients and 34 lean controls (Supplementary Table 2).

### 2.2 Platelet isolation and aggregation

Fresh blood samples (36 mL) were collected from obese patients and lean matched-controls in coagulation 3.2% sodium citrate Vacuette^®^ tubes and processed in less than 60 min. In order to obtain the platelet-rich plasma (PRP), blood was centrifuged for 20 min at 200 *g* at room temperature.

Washed platelets were isolated following a well-defined protocol that limits blood cells or plasma proteins contaminations [9]. Final washed platelet pellets were resuspended in HEPES-Tyrodes (134 mmol/L NaCl, 0.34 mmol/L Na_2_HPO_4_, 2.9 mmol/L KCl, 12 mmol/L NaHCO_3_, 20 mmol/L HEPES, 5 mmol/L glucose, 1 mmol/L MgCl_2_, pH 7.3) and allowed to rest for 30 min at room temperature.

For proteomic studies based on two-dimensional differential gel electrophoresis (2D-DIGE), concentrations of 8 × 10^8^ platelets/mL were lysed in a previously described NP-40-based lysis buffer and stored at −80 °C [10].

PRP or washed platelets (2.5 × 10^8^ platelets/mL in HEPES-Tyrodes) were used for aggregation studies. Aggregations were performed with 300 μL aliquots of PRP or washed platelets that were warmed at 37 °C for 4 min without stirring and for 1 min with constant stirring at 1200 rpm in a Chrono-log aggregometer, before stimulation with the corresponding agonists for 6 min.

In the case of PRP, stimulations were done with collagen-related peptide (CRP)-XL (0.1, 0.15 and 0.2 μg/mL), Horm collagen (0.5, 0.75 and 1 μg/mL), rhodocytin (25 and 50 nM), ADP (2 and 3 μM) and arachidonic acid (0.3 and 0.5 mM). Washed platelets were activated with collagen-related peptide (CRP)-XL (0.4, 0.5 and 1 μg/mL), Horm collagen (1, 2 and 3 μg/mL), rhodocytin (25, 50 and 100 nM) and thrombin (0.5 and 0.75 U/mL). Detailed information on the sources of agonists can be found in the Supplementary Materials.

### 2.3 2D-DIGE

For proteomic analysis, proteins were precipitated in 20% trichloroacetic acid in acetone, as previously described [9]. Protein pellets were resuspended in 60 μL DIGE buffer (65 mM CHAPS, 5 M urea, 2M thiourea, 0.15 M NDSB-256, 30 mM Tris, 1 mM sodium vanadate, 0.1 mM sodium fluoride, and 1 mM benzamidine). Protein quantitation was done with Coomassie Plus protein reagent (Thermo Scientific).

Six gels (technical replicates) were run in the experiment with a total of 150 μg of mixed labeled protein per gel. These protein mixtures contained 50 μg of protein from each sample (10 obese pooled and 10 lean pooled matched-controls) randomly labeled with 400 pmol minimal CyDye DIGE fluors (Cy3 and Cy5), and 50 μg of a pool of both conditions (25 μg from obese patients and 25 μg from lean controls) labeled with 400 pmol Cy2 (internal standard). Electrophoresis was done as indicated in the Supplementary Material. The first dimension was on immobilized pH gradient (IPG) strips 4–7, 24 cm (GE Healthcare). The second dimension was on 11% SDS-polyacrylamide gels. Following electrophoresis, gels were scanned directly in a Typhoon FLA 7000 scanner (GE Healthcare). Scanned images were processed with Progenesis SameSpots software (v 4.5) from Nonlinear Dynamics Ltd. (Newcastle, UK) in order to find real differences between conditions. Further information on the 2D-DIGE and image analysis protocols can be found in the Supplementary Materials.

### 2.4 Mass spectrometric analysis

Spots of interest were excised manually from the gels and in-gel digested with trypsin as previously indicated [11]. Most of the identifications were by LC–MS/MS on an EASY-nLC (Proxeon, Bruker Daltonics) and a Bruker Amazon ETD ion trap. Remaining identifications were by MALDI-TOF(/TOF), in a 4800 Plus MALDI-TOF/TOF analyzer (Applied Biosystems). Database search was performed with the Mascot Version 2.3.0 search tool (Matrix Science, London, United Kingdom) screening SwissProt. Precise details on the MS protocols and database searching parameters can be found in the Supplementary Methods.

### 2.5 Systems biology

Ingenuity Pathways Analysis (IPA) software (Ingenuity Systems, CA, USA) was used to investigate possible interactions among all the identified proteins. STRING v10 software [12] was also used to predicted protein-protein interactions and to know the biological process, molecular functions and cellular components where the differential proteins were involved.

### 2.6 Western blotting

Western blotting was performed to analyze individual samples (biological replicates) for validation purposes. 11% SDS-PAGE gels were used, loading 10 μg of protein per lane. In some cases, 2D-western blotting validations were carried out, using IPG strips pI 4–7, 7 cm (GE Healthcare). In that case, 30 μg of protein were loaded per strip; obese and lean samples were analyzed in parallel. IEF (first dimension) was for 11Kvh; second dimension was on 11% SDS-PAGE gels.

Following electrophoresis, proteins were transferred onto polyvinylidene difluoride (PVDF) membranes. Membranes were blocked in 5% BSA in TBS-T (20 mM Tris-HCl, (pH 7.6), 150 mM NaCl and 0.1% Tween 20) overnight at 4 °C and incubated for 90 min at room temperature with the following primary antibodies: rabbit anti-phospho-SRC (Tyr418) (Invitrogen), dilution 1:1000; rabbit anti-SRC pan (Invitrogen), dilution 1:1000; mouse anti-FIB (sc-69775, Santa Cruz), dilution 1:1000; mouse anti-PLCγ2 (sc-5283, Santa Cruz), dilution 1:1000; rabbit anti-GAPDH (SIGMA), dilution 1:5000; and anti-G6f (1:1000), produced by CovalAB UK (Cambridge, UK). Following washes in TBS-T, membranes were exposed to horseradish peroxidase–labeled goat anti-rabbit, or goat anti-mouse antibodies (dilution 1:5000) (Pierce, Rockford, IL), and processed using an enhanced-chemiluminiscence system (ECL, Pierce, Rockford, USA).

### 2.7 Immunoprecipitation

Basal and activated platelets (8 × 10^8^platelets/mL, 500 μL per immunoprecipitation; activations with CRP-XL 1 μg/mL, 90sec) were lysed with NP40-based lysis buffer, as indicated in Supplementary Material. Activations were under non-aggregating conditions in the presence of integrilin (9 μM). Phosphotyrosine (*p*-Tyr) immunoprecipitations were done with 5 μg of agarose-conjugated 4G10 monoclonal anti-phosphotyrosine antibody (EMD Millipore Corporation, Billerica, MA, USA) per sample, as previously shown [13]. Further details on the protocol can be found in Supplementary Material.

Immunoprecipitations from twelve obese patients and ten lean matched-controls were loaded independently, and proteins resolved on 4–12% NuPAGE Bis-Tris gradient gels (Invitrogen, Carlsbad, CA, USA). Following the electrophoresis, proteins were transferred onto polyvinylidene difluoride (PVDF) membranes (GE Healthcare) and incubated with primary antibodies (PLCγ2 and G6F) as describe above.

### 2.8 Flow cytometry

To measure GPVI dimer, GPVI total and P-selectin, 10 μL of platelet solution, either 5-fold diluted whole blood or washed platelets (5 × 10^7^ cells/mL), was mixed with 10 μL of 204-11 Fab (GPVI-dimer-specific; 5 μg/mL, final), 1G5 (pan GPVI; 10 μg/mL, final) (Biocytex, France) and CD62 (10 μg/mL, final) (ABCAM, UK) and incubated for 10 min. For all of them, ALEXA 488 anti-mouse F(ab)2 antibody (50 μg/mL, final) (Jackson ImmunoResearch) was added as the secondary antibody and incubated for 10 min. In individual Eppendorf tubes each reaction mixture was diluted with 0.200 mL of diluent (Beckman Coulter, USA), and then antibody binding was measured by an Accuri C6 flow cytometer (BD Biosciences). Platelet binding to appropriate controls, mouse Fab (Jackson ImmunoResearch) when the primary antibody was 204-11 Fab, IgG2A when it was 1G5 and IgG1 when it was CD62, was determined. The test was run in triplicate and the average of mean fluorescence intensity was measured.

### 2.9 Statistical analysis

Categorical variables from obese patients and lean matched-controls are expressed as percentages and were compared using the Fisher exact test. Continuous variables are expressed as the median ± SD, and were compared by the Mann-Whitney test. Correlations analyses were done using the Spearman's test.

As indicated above, the differential proteomic analysis was done analyzing all the spots between obese patients and lean matched-controls; for a given spot, the probability value was calculated using the quantified and normalized volumes for the matched spot. All probability values were calculated using 1-way ANOVA analysis and values of *p* < 0.05 were considered statistically significant.

For 1D-western blotting validation studies, protein bands intensity was quantified by densitometry using ImageJ (National Institute of Health, Bethesda, MD, USA) version 1.47. All analyses were performed using IBM SPSS Statistics 20 software for Windows (IBM, Armonk, NY, USA).

## 3 Results

### 3.1 Patients’ characteristics

Thirty-four patients admitted to the hospital with a diagnosis of severe “healthy” obesity (BMI ≥ 40) and 34 age- and gender-matched lean healthy controls were included in the study (Supplementary Table 2). From those, a cohort of 10 obese patients and 10 matched-controls were included in the proteomic analysis (Supplementary Table 1), with only 1 diabetic patient. Apart from the diabetic patient, HBA1c levels were within the normal range (below 6.5%).

Significant clinical differences between groups were observed in the BMI and leukocytes count parameters, as expected. Unexpectedly, there was also an increase in MPV in the lean group, when analyzing all patients without subgroup divisions, although all values fell within the normal physiological range (Supplementary Table 2).

### 3.2 Differential platelet proteome analysis: most up-regulated proteins from obese patients are related to platelet activation and aggregation

**Fig. 1:**
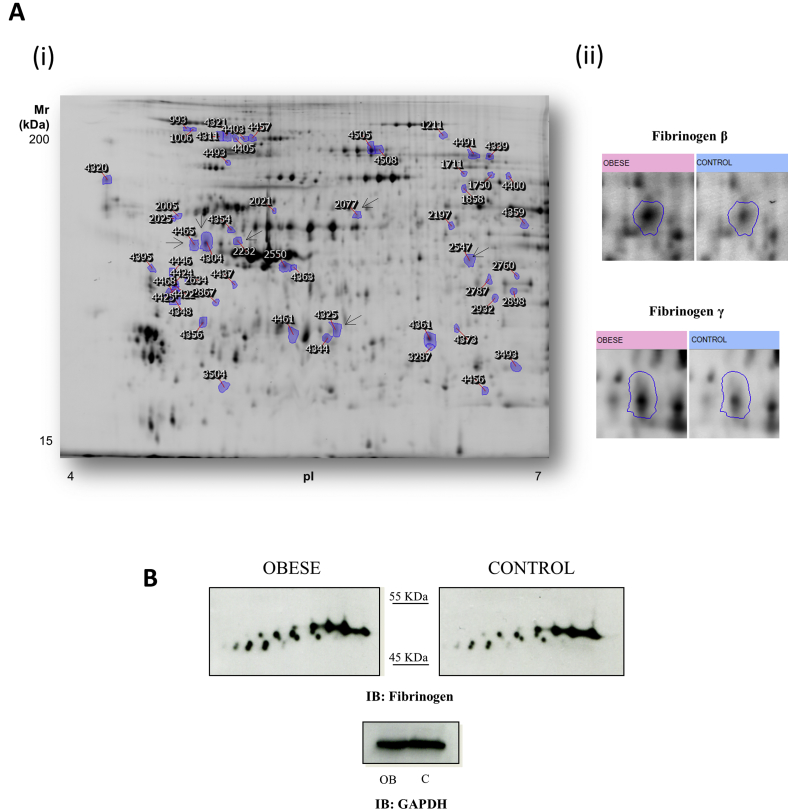
2D-DIGE-based differential proteome analysis of platelets from obese and lean individuals: up-regulation of fibrinogen features in obese patients. (A-i) Representative image of the analysis in grey scale. The figure shows the location on the 2D gels of those spots that are differentially regulated when comparing obese patients and lean matched-controls. Protein identifications are shown by the identification numbers in Supplementary Tables 3 and 4. Spots corresponding to fibrinogen are indicated with a black arrow. (A-ii) Enlarged images of representative fibrinogen spots found to be up-regulated in obese patients by proteomics. (B) Representative 2D-Western blot images of fibrinogen on pools of samples from obese-patients’ platelets and lean matched-controls (n = 22 per group). GAPDH was used as loading control (1D-western blot). Western blot experiments were run in triplicate. OB: severe obese patients; C: lean matched-controls; IB: immunoblot.

The 2D-DIGE-based proteomic analysis focussed on the pI 4-7 range, where most of the platelet proteome is located [14]. In total, 1895 protein spots were detected per gel, 55 of which were differentially regulated between obese and lean groups (fold change cut-off ≥ 1.2; *p* < 0.05) (Fig. 1A). Out of those 55 differences, 25 were up-regulated in obese patients whereas 30 were down-regulated. A principal components analysis (PCA) showed a very good separation between groups based on the proteome profile (data not shown). We could successfully identify 35 protein features by MS which corresponded to 33 different ORFs (Supplementary Tables 3 and 4).

Regarding protein function, 17 of the identified proteins play an important role in stress response, including oxidative stress (e.g. heat shock protein beta-1) and inflammatory response (e.g. zyxin and lysozyme C). Another important group represented is related to maintenance of hemostasis, platelet aggregation and degranulation (e.g. integrin αIIb, fibrinogen). In fact, STRING v10.5 software [12] indicated that 50% of the obese up-regulated proteins are involved in platelet activation and aggregation (e.g. actin, actinin-1, fibrinogen, thrombospondin-1 (TSP1), integrin αIIb). Moreover, Ingenuity Pathways Analysis software (IPA, Ingenuity Systems, www.ingenuity.com) identified integrin signalling as the most affected pathway related to the proteins identified. In addition, the top molecular and cellular functions related to the differentially regulated proteins identified are aggregation of blood platelets and thrombus formation. Moreover, most of the altered proteins identified are part of a common network related to cell-to-cell signaling, hematological system development and function, and inflammatory response (Supplementary Fig. 1).

One of the proteins up-regulated in platelets from obese patients was fibrinogen, previously reported in the platelet proteome [14]. Our proteomic analysis showed the presence of this protein in 7 differentially regulated spots: two of them corresponded to the β-chain and 5 to the γ-chain. All of these spots were found to be up-regulated in platelets from obese patients. Fibrinogen levels were validated by 2D Western blotting on pools (22 severe obese patients and 22 lean controls). In line with the proteomics results, a series of fibrinogen spots appear up-regulated in obese patients’ samples (Fig. 1B).

### 3.3 Platelet aggregation in response to GPVI stimulation is increased in obese patients compared to lean matched-controls

In parallel to the proteomic studies, we carried out platelet aggregation-based functional analysis.

Platelets were activated with various agonists: GPVI ligands, such as, CRP-XL and collagen at different doses and conditions (PRP and washed platelets), rhodocytin, arachidonic acid, ADP and thrombin (Supplementary Tables 5 and 6).

**Fig. 2:**
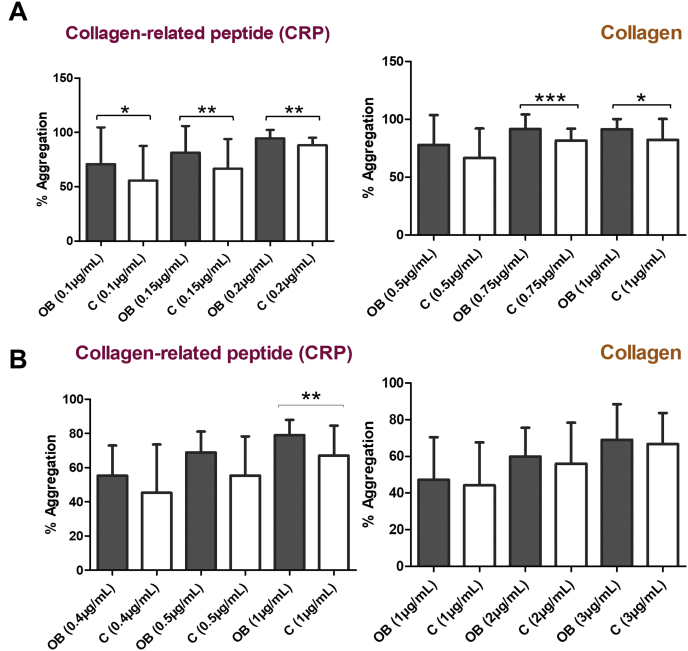
Platelet aggregation in response to GPVI activation is increased in obese patients compared to lean matched-controls. PRP and washed platelets were stimulated with different GPVI agonists. (A) PRP was stimulated with CRP-XL (0.1, 0.15 or 0.2 μg/mL) or Horm collagen (0.5, 0.75 or 1 μg/mL) to trigger aggregation. (B) Washed platelets were stimulated with CRP-XL (0.4, 0.5 or 1 μg/mL) and Horm collagen (1, 2 or 3 μg/mL). Results are presented as the mean ± SD; *p < 0.05, **p < 0.01, ***p < 0.001. *p*-values and cohort data are shown in Supplementary Table 5. OB: severe obese patients; C: lean-matched controls.

Platelet aggregation following activation of GPVI was higher in severe obese patients. As expected, there were certain differences among conditions (PRP or washed platelets) (Fig. 2).

However, no significant differences in platelet aggregation between obese and lean matched-controls were observed following activation with rhodocytin (CLEC-2 agonist); or with non-SFKs-mediated signalling pathways agonists, such as ADP, arachidonic acid and thrombin (Supplementary Fig. 2).

These aggregation studies showed that obese platelets are hyper-reactive in response to collagen and CRP, pointing towards GPVI signalling as one of the altered pathways in obesity.

### 3.4 Active Src (pTyr418) is up-regulated in platelets from obese patients

The proteomic identification of up-regulated proteins such as integrin αIIb, fibrinogen, actinin 1 and vinculin and the hyper-reactivation of platelets in response to CRP-XL and collagen in obese patients points towards a possible alteration of SFKs-mediated pathways.

Src is a 60-kDa nonreceptor tyrosine kinase involved in transmitting activation signals from a diverse repertoire of platelet surface receptors, including integrin αIIbβ3, the immunoreceptor tyrosine–based activation motif–containing collagen receptor complex Glycoprotein VI (GPVI)-FcRγ-chain, and the von Willebrand factor receptor complex GPIb-IX-V, which are essential for thrombus growth and stability [15].

**Fig. 3:**
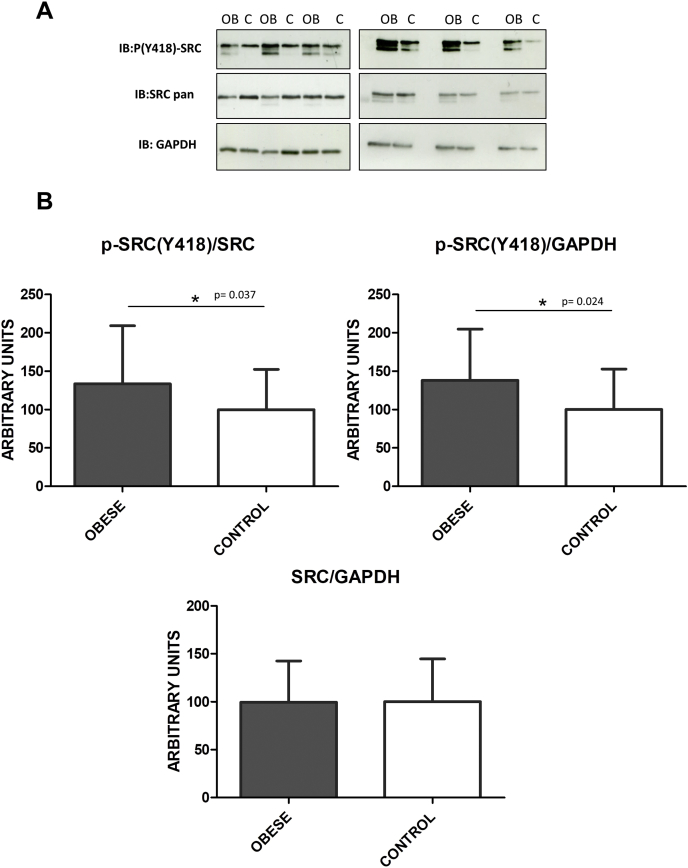
P-Src (pTyr 418) levels are augmented in platelets from obese patients. (A) 1D-Western blot analysis of Src-pTyr418, Src pan and GAPDH protein expression levels in platelets from individual samples (29 obese patients and 29 lean matched-controls). Images are representative of the results obtained and show samples distributed in two gels. (B) Densitometry graphs showing the mean values ± SD of Src (Tyr 418) of 30 obese patients and their lean matched-controls corrected by Src and the loading control (GAPDH), *p*-values are 0.037 and 0.024 respectively. Moreover, densitometry graph showing the mean values ± SD of Src corrected by the loading control (GAPDH) indicates no differences between obese and control groups (*p*-value = 0.97). OB: severe obese patients; C: lean matched-controls; IB: immunoblot; *p < 0.05.

Src and other members of the SFKs are tightly regulated by tyrosine phosphorylation. Full catalytic activity of Src requires phosphorylation of tyrosine 418, which is located in the catalytic domain. By using a specific anti-Src (pTyr418) antibody, we demonstrated that the active form of Src is up-regulated in platelets from a cohort of 29 “healthy” obese patients with BMI ≥ 40 compared to matched lean healthy controls (Fig. 3).

### 3.5 PLCγ2 is hyper-phosphorylated in platelets from obese patients following CRP-XL stimulation

**Fig. 4:**
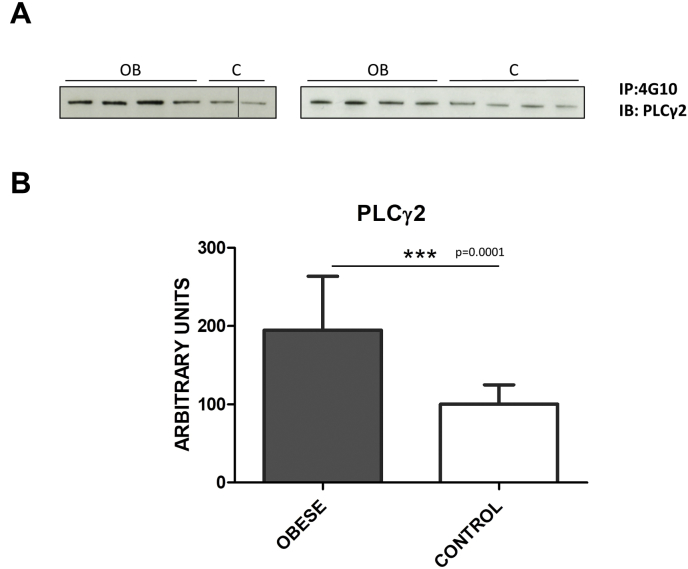
PLCγ2 phosphorylation levels are increased in platelets from obese patients following CRP-XL stimulation. Immunoblot analysis of PLCɣ2 following immunoprecipitation with the anti-phosphorylation 4G10 mAB. Severe obese and lean matched controls are compared using 4 × 10^8^ platelets per immunoprecipitation. All samples were stimulated with CRP (1 μg/mL; 90 s), as indicated in the Methods section. (A) Representative images corresponding to individual patients are shown. Samples were distributed in two gels; a vertical line indicates a failed well. (B) Densitometry graph showing the mean values ± SD for all patients (severe obese: 12; lean matched controls: 10). Significant differences are indicated: ***p < 0.0001. IP: immunoprecipitation; IB: immunoblot.

To complement the data shown above, we analyzed a sub-group of patients to clarify alterations in GPVI signalling following CRP-XL stimulation. We chose PLCγ2 as a key protein in GPVI signalling that we previously observed to be altered following CRP-XL stimulation in platelets from ST-elevation myocardial infarction (STEMI) patients [13]. After immunoprecipitation with the 4G10 antiphosphotyrosine antibody, we saw an increase of PLCγ2 tyr phosphorylation in the obese group compared to lean matched-controls, which suggests a hyperactivation of GPVI signalling in line with the results shown above (Fig. 4).

However, we found no significant differences in G6F, as opposed to the STEMI study [13] (data not shown).

### 3.6 Higher expression levels of total GPVI and GPVI dimer in the surface of platelet from obese patients

**Fig. 5:**
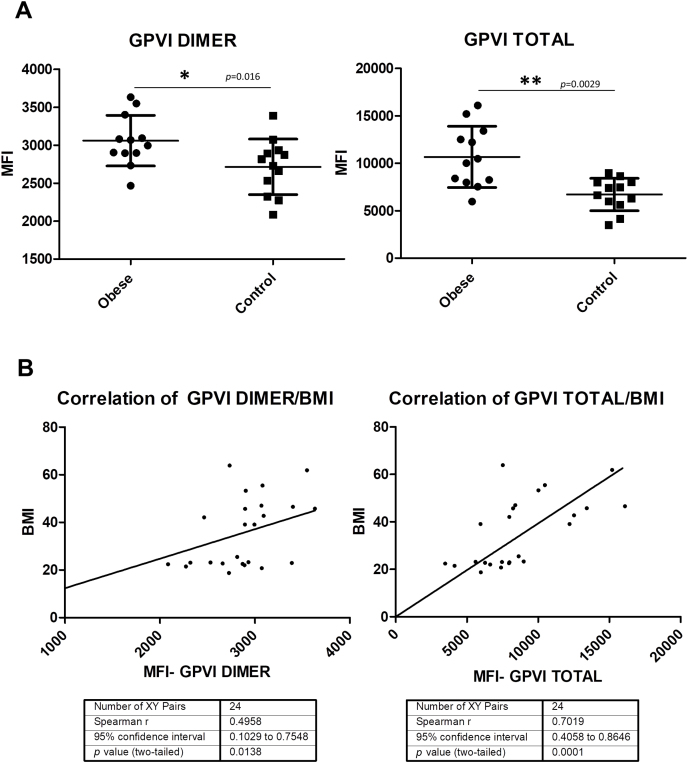
Higher expression levels of GPVI dimer and total GPVI in the surface of platelets from obese patients, with a positive correlation with BMI. The study was performed with a subgroup of patients (12 severe obese patients and their 12 lean matched-controls) and measured in triplicate. (A) GPVI dimer and total levels of GPVI are increased in the obese group. Fluorescence values are shown as mean ± SD. Significant differences are indicated as follow: *p < 0.05, **p < 0.01. (B) Positive correlation between BMI and total GPVI and GPVI dimer was found using Spearman's test.

Flow cytometry was used to mechanistically understand the aggregation and biochemical data that pointed towards alterations in GPVI signalling in obese patients. For that reason, we analyzed the expression of GPVI in whole blood from 12 “healthy” severe obese and 12 lean matched-controls. By using specific antibodies, we studied the platelet surface expressions levels of total GPVI and the dimer form (GPVI-dimer) [16]. We demonstrated higher expression levels of both of them in obese patients, which could explain the observed hyperactivation of GPVI signalling (Fig. 5A). In addition, we found a positive correlation between total GPVI, GPVI-dimer and BMI in all participants, of the study (Fig. 5B). Additionally, we investigated another group of 11 healthy individuals with BMI between 26 and 35 (overweight and grade I obesity). Interestingly, we also found higher expression levels of GPVI (total and dimer) in this group compared to lean individuals (Supplementary Fig. 3A). When integrating these data with those obtained for severe obese patients (Fig. 5), the positive correlation with BMI is maintained (Supplementary Fig. 3B).

Finally, no significant differences between severe obese patients and lean matched-controls were found when levels of p-selectin were measured in washed platelets although there is a tendency to increased levels in the obese group (not shown).

## 4 Discussion

The principal findings of the present study are: (i) proteomics alterations in proteins related to platelet signalling - including integrin signalling - in obese patients together with increased levels of fibrinogen, the latter validated by immunoblotting; (ii) the identification of increased aggregation levels of platelets from obese patients in response to CRP-XL and Horm collagen, pointing towards GPVI signalling as one of the altered pathways in obesity; (iii) there is a higher expression of the active form of Src (pTyr418) – essential for GPVI and integrin signalling - in platelets from obese patients compared to lean individuals, linking proteomic and aggregation data; (iv) CRP-activated platelets present higher levels of tyrosine phosphorylated PLCγ2 in obese patients, which is in agreement with the previous data; (v) flow cytometry results show higher surface expression levels of total GPVI and GPVI-dimer in platelets from obese patients correlating positively with the BMI; this latter point provides a mechanistic explanation for the GPVI signalling data.

Focusing on the proteomic analysis, most of the proteins identified in the present study are interconnected in a common network that primarily involves integrin and actin cytoskeleton signalling, processes closely related to platelet aggregation and activation. Moreover, Ingenuity Pathways Analysis software revealed inflammatory response, thrombosis, and CVD as the most affected diseases related to the differentially regulated proteins identified.

It is noteworthy that some of the proteins identified such as TSP1 and fibrinogen were previously found to be involved in obesity [[17], [18], [19], [20]]. TSP1 is a major component of platelet alpha granules although it exists as both a component of the extracellular matrix and as a soluble molecule found in various body fluids [19]. In fact, TSP1 has recently been highlighted as a potential mediator of insulin resistance and adipose inflammation in obesity [18].

In relation to fibrinogen, we focused on this protein for validation studies because it is highly represented in the analysis and has been described as a potential biomarker for obesity and CVD [20,21]. Fibrinogen is a precursor of the clot structural protein fibrin. It has a multimeric structure consisting of α, β and γ polypeptides. Two of these polypeptides (β and γ) were detected in the present study. The γ chain is of particular interest because it contains multiple epitopes that interact with integrins, and plays a critical role in platelet aggregation and inflammation [22]. In fact, higher levels of fibrinogen γ have been repeatedly reported in CVD and obesity [[23], [24], [25]].

We identified the γ-isoform in 5 spots that were all up-regulated in obese patients. This result was confirmed by western blotting. The up-regulation of fibrinogen in obese platelets is consistent with the increased levels previously found at circulating level in obese patients [26,27] which could promote a hype-reactive state of different signalling pathways such as those related to integrin αIIbβ3. Moreover, a recent study with mice carrying genetically imposed deficiencies or functional mutations in fibrinogen showed that fibrinogen drives high-fat diet-induced obesity. This study concludes that the thrombin/fibrin(ogen) axis is not only a critical trigger of high fat diet-mediated systemic inflammation, but a pharmacologically and genetically adjustable driver of obesity [28]. In addition, it is also relevant to point out that GPVI was recently reported as a fibrin and fibrinogen receptor [[29], [30], [31]]. All the above data is consistent with the potential alterations in integrin αIIbβ3 and GPVI signalling identified in the present study.

GPVI and αIIbβ3 platelet stimulation leads to activation of Src through autophosphorylation at position Tyr418 [14,32]. Interestingly, we demonstrate that the active form of Src (pTyr418) is up-regulated in platelets from obese patients, suggesting a potential hyperactivation state of those pathways, such as GPVI, where SFKs are essential. In line with the latter, we also showed that PLCγ2, a key protein of the signalling pathway, was hyper-phosphorylated in the obese group following CRP-XL platelet stimulation. This suggests a higher activated state of GPVI signalling in obese patients, as we previously reported for STEMI patients [13].

Additionally, our aggregation assays provided further evidence that there is an alteration of GPVI signalling in platelets from obese patients. Platelets from the obese individuals showed enhanced aggregation in response to collagen and the GPVI-specific agonist CRP-XL compared to lean matched-controls, suggesting a higher activation of this signalling pathway in obese patients. Aggregation data are in line with the biochemical results shown above.

There are various explanations for the GPVI signalling data discussed above. High levels of fibrinogen in plasma could interact with GPVI receptor enhancing the pathway activation [33]. However, another possibility for our results could be increased surface expression levels of platelet GPVI (total or the dimeric form) in obese patients as it has already been shown in other diseases such as ischemic stroke [31,34]. Both possibilities could co-exist.

To clarify the results mechanistically, we performed a FACs assay where we demonstrated higher expression levels of the dimer form of GPVI and total GPVI in obese patients, which must be directly linked to the higher activation levels of GPVI signalling and GPVI-mediated aggregation observed in obese patients.

Finally, it is necessary to point out that this is a complex study and is not free of some limitations: firstly, sample limitation was an issue, somehow unavoidable when dealing with clinical samples; this was the reason for pooling samples for the 2D-DIGE-proteomics and 2D-western blotting analyses. Secondly, the variety of participants and where they could be recruited (severe obese patients at the hospital, overweight and lean volunteers at the Universidade de Santiago Health Service) made difficult to obtain the same clinical-biochemical parameters for all of them; nevertheless, the most relevant ones could be obtained and shown in the clinical tables.

In conclusion, the present study is the first to analyze in detail the platelet proteome of obese patients. It provides novel information on platelet proteome changes related to obesity and, in combination with functional and biochemical assays, highlights those platelet signalling pathways altered with higher probability in this pathology, such as SFK-mediated pathways. Fibrinogen and the active form of the tyrosine kinase Src were found to be up-regulated in platelets from obese patients. Moreover, we show an up-regulation of GPVI signalling and GPVI platelet surface expression in obese patients. Our results suggest that obese patients present an atherothrombotic risk that should be considered, and highlight the relevance of considering novel antithrombotic drug targets in these patients, such as GPVI. We hope that our study will open new lines of investigation to further explore the pathophysiological role of platelets in obesity and find novel therapeutic targets and platelet-related biomarkers for this type of patient.

## Conflicts of interest

The authors declare they do not have anything to disclose regarding conflicts of interest with respect to this manuscript.

## Financial support

This work was supported by the Spanish Ministry of Economy and Competitiveness (MINECO) [grant No. SAF2016-79662-R], co-funded by the European Regional Development Fund (ERDF). Financial support from the Consellería de Cultura, Educación e Ordenación Universitaria, Xunta de Galicia (Centro Singular de investigación de Galicia accreditation 2016–2019, ED431G/05), and the European Regional Development Fund (ERDF) is also gratefully acknowledged. SMJ and RWF were supported by British Heart Foundation, grants SP/10/011/28199 and RG/15/4/31268.

## Author contributions

M.N. Barrachina performed research, analyzed data and wrote the paper. A.M. Sueiro supervised clinical work and analyzed data. F.F. Casanueva supervised clinical work. I. Izquierdo and L. Hermida-Nogueira performed experiments; E. Guitián performed mass spectrometry analyses; R. Farndale, M. Moroi and S.M. Jung contributed with key reagents and analytical tools. M. Pardo contributed to the study design and revised the manuscript. A. García designed the study, contributed with key reagents and analytical tools, and wrote the paper.
