# Supplementary material for: GPVI surface expression and signalling pathway activation are increased in platelets from obese patients: Elucidating potential anti-atherothrombotic targets in obesity
Source: Atherosclerosis. 2019 Feb;281:62–70. doi: 10.1016/j.atherosclerosis.2018.12.023 (PMC6403451; doi:10.1016/j.atherosclerosis.2018.12.023)
Supplement: Supplementary data [file mmc1.pdf]

SUPPLEMENTARY MATERIAL

Platelet agonists

The following agonists were used: Crosslinked collagen-related peptide (CRP), with the sequence Gly-Cys-Hyp-(Gly-Pro-Hyp)$_{10}$-Gly-Cys-Hyp-Gly-NH2, was provided by Dr. Richard W. Farndale, from the University of Cambridge (UK). Collagen Reagent HORM® Suspension (KRH) was purchased from Takeda Austria GmbH (Austria). Thrombin and ADP were purchased from Sigma (Sigma-Aldrich, St. Louis, MO) and arachidonic acid from Cayman Chemical (Michigan, USA).

Rhodocytin was provided by Johannes A. Eble, from Center for Molecular Medicine, Excellence Cluster Cardio-Pulmonary System, Frankfurt University Hospital, Frankfurt am Main, Germany.

Supplementary Methods

2D-DIGE

Six gels (technical replicates) were run in the experiment with a total of 150 µg of mixed labelled protein per gel. These protein mixtures contained 50 µg of protein from each sample (10 obese pooled and 10 lean pooled matched-controls) randomly labeled with 400 pmol minimal CyDye DIGE fluors (Cy3 and Cy5), and 50 µg of a pool of both conditions (25 µg from obese patients and 25 µg from lean controls) labeled with 400 pmol Cy2 (internal standard). Labelling was performed for 30 min on ice in the dark. The reaction was stopped with 1 µl of 10 mM lysine acting for 10 min on ice in the dark. After labelling step, the three samples were pooled and an equal volume of 2× sample buffer was added (65 mM CHAPS, 2 M thiourea, 5 M urea, 0.15 M NDSB-

256, 130 mM DTT, 4 mM tributylphosphine, 1 mM sodium vanadate, 0.1 mM sodium fluoride, and 1 mM benzamidine).

After mixing, the tube was left for 10 min on ice in the dark. For reswelling, samples were diluted up to a total of 500 µl of 2D Sample buffer (5 M urea, 2 M thiourea, 2 mM tributylphosphine, 65 mM DTT, 65 mM CHAPS, 0.15 M NDSB-256, 1 mM sodium vanadate, 0.1 mM sodium fluoride, and 1 mM benzamidine, final concentration), and ampholytes (Servalyt 4–7) were added to a final concentration of 1.6% (v/v). IPG strips were rehydrated with the samples for 16 h in the dark. Isoelectric focusing (IEF) was run on 24 cm, pH 4–7 IPG strips (GE Healthcare) powered by a Multiphor II (GE Healthcare) for 64.9 kVh at 17 °C.

Following the first dimension, IPG strips were immediately equilibrated for 15 min in reduction buffer (6 M urea, 50 mM tris pH 8.8, 30% glycerol, 2% w/v SDS, 65 mM DTT and traces of bromophenol blue) and then for 15 min in alkylation buffer (6 M urea, 50 mM tris pH 8.8, 30% glycerol, 2% w/v SDS, 135 mM iodoacetamide and traces of bromophenol blue) with gentle agitation; all steps in the dark. IPG strips were washed out with ultrapure water and placed on top of the second dimension gels, embedded with 0.5% melted agarose.

Proteins were separated in the second dimension by SDS-polyacrylamide gel electrophoresis (PAGE) on 11% polyacrylamide gels at run conditions of 10°C, 20 mA per gel for 1 h, followed by 40 mA per gel for 4 h by using an Ettan Dalt 6 system (GE Healthcare). Following electrophoresis gels were scanned directly in a Typhoon FLA 7000 scanner (GE Healthcare). After imaging, gels were fixed in 10% methanol/ 7% acetic acid for 1 hour, and stained overnight with Sypro Ruby fluorescent dye for spot picking.

Differential Image Analysis

Scanned images were processed with Progenesis SameSpots software (v 4.5) from Nonlinear Dynamics Ltd. (Newcastle, UK) in order to find real differences between both conditions of study. Both manual and automatic alignment was used to align the images. SameSpots detects the spots simultaneously across all images generating a master gel list containing all the features. All gels were compared with each other and fold values as well as P-values of all spots were calculated by SamesSpots software using one way ANOVA analysis. Differential expression of a protein present in the gels was considered significant when the fold change was at least 1.2 and the P-value was below 0.05.

Mass Spectrometric Analysis

Protein identifications were by LC-MS/MS. Digested peptide mixtures dissolved in 0.1% formic acid were separated in an EASY-nLC (Proxeon, Bruker Daltonik GmbH) with a reverse phase nanocolumn (Easy column SC200) from Proxeon (see below for more details).

LC parameters for separation of tryptic peptides

LC settings

LC system	Easy-nLC PROXEON
Trap column	Easy-column SC001 L 2 cm, ID 100 um, 5 um, 120 A, C18-A1 from Proxeon
Analytical column	Easy column SC200 C18 3µm 120A 360 µm OD/75µm ID, L=10cm) from Proxeon
Flow rate	300 nl/min

Eluents	A:0.1% FA in water B:0.1%FA in ACN
Gradient	5% (t=0 min), 35% (t=32 min), 50% (t=37 min), 100% (t=38min)

Ionized peptides were analyzed in a CID-ETD ion trap mass spectrometer (Bruker Daltonics), equipped with a Nanosprayer ionization source that was used for data-dependent MS/MS experiments. Spectra were acquired in Enhanced Resolution mode. Further acquisition parameters are listed in below.

MS and MS/MS settings used for acquisition with the Amazon ETD

Acquisition parameters

Source	Nanosprayer®
MS settings:	
Scan mode	Enhanced resolution mode (8,100 m/z s^{-1})
Scan range	50-3000 Da
Spectra averages	5 (Rolling averaging: 1)
MS/MS settings:	
Scan mode	UltraScan
Scan range	100-3,000 m/z
No. of precursor ions	3 (active exclusion after 1 spectrum, release after 0.2 min; reconsider precursor, if current intensity/previous intensity >1%
Isolation width	4 m/z
Spectral averages	2
Fragmentation amplitude (CID)	30-300%
Fragmentation time (ETD)	100 ms

Automated analysis of mass data was achieved by Data Analysis 4.0 and BioTools 3.2 from Bruker Daltonik GmbH. Database search was performed with the Mascot v2.3.0 search tool (Matrix Science, London, UK) screening SwissProt (SwissProt_2016_09.fasta). Searches were restricted to human taxonomy allowing carbamidomethyl cysteine as a fixed modification and oxidized methionine as potential variable modification. Both the precursor mass tolerance and the MS/MS tolerance were set at 0.3 and 0.4 Da, respectively, allowing 1 missed tryptic cleavage site. All spectra and database results were manually inspected in detail using the above software, especially in the case of identifications based on one peptide hit. For the latter, positive identification by MS was only accepted when more than 50% y-ions (CID fragmentation) or z-ions (ETD fragmentation) were obtained for a peptide comprising at least eight amino acids long and no missed tryptic cleavage site. Positive hits corresponded to Mascot scores > 40 plus the fulfillment of the above criteria.

For MALDI analysis, dried peptides were dissolved in 4 µL of 0.5% formic acid. Equal volumes (0.5 µL) of peptide and matrix solution (3 mg alpha-cyano-4-hydroxycinnamic acid (α-CHCA) dissolved in 1 mL of 50% acetonitrile in 0.1% trifluoroacetic acid) were deposited using the thin layer method, onto a 384 Opti-TOF 123x81 mm MALDI plate (Applied Biosystems) and allowed to dry at room temperature. Mass spectrometric data were obtained in an automated analysis loop using a 4800 MALDI-TOF/ TOF analyzer (Applied Biosystems). Spectra were acquired in the reflector positive-ion mode with a Nd:YAG, 355nm wavelength laser, at 200 Hz laser frequency, and 1000 to 2000 individual spectra were averaged. The experiments were acquired uniform with fixed laser intensity. All MSMS spectra were performed by

selecting the precursors with a relative resolution of 300 (FWHM) and metastable suppression.

Automated analysis of mass data was achieved by using the 4000 Series Explorer Software V3.5 (Applied Biosystems). Internal calibration of MALDI-TOF mass spectra was performed using two trypsin autolysis ions with m/z = 842.510 and m/z = 2211.105. For MALDI-MS/MS, calibrations were performed with fragment ion spectra obtained for Angiotensin II (peptide mix, calibration standard, Bruker). MS and MS/MS spectra data were combined through the Protein Pilot Explorer Software v4.5. Database search was performed with the Mascot v2.1 search tool (Matrix Science, London, UK) screening SwissProt (release version 2016-05; February, 551193 entries). Searches were restricted to Human taxonomy allowing carbamidomethyl cysteine as a fixed modification and oxidized methionine as potential variable modification. Both the precursor mass tolerance and the MS/MS tolerance were set at 100 ppm and 0.3 Da, respectively, allowing 1 missed tryptic cleavage site. MALDI-MS(/MS) spectra and database search results were manually inspected in detail using the previous software. For combined MS and MS/MS data, identifications were accepted when Confidence Interval (C.I.%) of Protein Pilot software was 95% or higher. Since Protein Scores and Ion Scores from different searches cannot be directly compared, Protein Pilot software calculates this C.I.% in order to combine results from MS and MS/MS database searches. This coefficient value means that the probability that the observed match is a random event is lower than 5%. For PMF spectra, identifications were also accepted when (C.I.%) of Protein Pilot software was 99% or higher.

Immunoprecipitation

Basal and activated platelets (8×10^8platelets/mL, 500 µL per immunoprecipitation; activations with CRP-XL 1µg/mL, 90sec) were lysed with 500 µL NP40-based lysis buffer, (0.3M Sodium Chloride, 20mM Tris, 2mM EGTA, 2mM EDTA, 2% (v/v) NP-40, pH 7.5). Activations were under non-aggregating conditions in the presence of integrilin (9 µM). For phosphotyrosine (p-Tyr) immunoprecipitations, 5 µg of agarose-conjugated 4G10 monoclonal anti-phosphotyrosine antibody (EMD Millipore Corporation, Billerica, MA, USA) were added to the lysates per immunoprecipitation and samples rotated overnight at 4 °C. Before the addition of the antibodies, samples were precleared with 25 µL of Protein A-Sepharose (50% w/v in TBS-T (20mM Tris-HCl (pH 7.6), 137mM NaCl, and 0.1% v/v Tween 20)) at 4 °C for 60 minutes with end-over-end mixing.

After immunoprecipitations, proteins were eluted from the beads in 2X Laemmli sample buffer (4% w/v SDS, 10% v/v 2-mercaptoethanol, 20% v/v glycerol, 50 mM Tris, pH 6.8) and resolved on 4–12% NuPAGE Bis-Tris gradient gels (Invitrogen, Carlsbad, CA, USA) for western blotting.

Supplementary Table 1. Clinical characteristics of obese and lean-matched control- proteomics study.

Variable	Obese patients (N =10)	Lean-matched controls (N =10)	P value
Age (years)	33.50 ± SD 11.90	33.50 ± SD 11.30	1
Females (%)	90%	90%	1
BMI***	46.30 ± SD 6.07	21.83 ± SD 1.96	<0.001
Diabetes (%)	10%	0%	-
HbA1c (%)	5.39 ± SD 0.45 (n=7)	-	-
Laboratory Measurements			
Hemoglobin (g/dl)	13.46 ± SD 0.71	13.50 ± SD 0.85	0.939
Leukocytes/µL*	8.70 ± SD 2.62	7.75 ± SD 6.58	0.028
Platelets/µL	255400 ± SD 60110	242400 ± SD 60120	0.762
Mean Platelet Volume (fL)*	10.07 ± SD 1.60	8.70 ± SD 1.49	0.037
Glucose (mg/dl)	83.10 ± SD 13.33	83.60 ± SD 8.04	0.570
Creatinin (mg/dl)	1 ± SD 0.0	0.72 ± SD 0.09	0.058
Cholesterol (mg/dl)	175.10 ± SD 24.48	194.50 ± SD 37.88	0.211
Chronic Treatments			
Antiplatelets (%)	0%	0%	
ACE Inhibitors (%)	0%	0%	
Benzodiazepines (%)	0%	0%	
Celecoxib (%)	0%	0%	
Statins (%)	0%	0%	
Antidepressants (%)	0%	0%	
Muscle relaxant (%)	0%	0%	

a. Data are presented as the median ± SD or percentage of patients. * $p<0.05$.

b. Patients with glucose levels below 80 mg/dL were not requested HbA1c measuring.

Supplementary Table 2. Clinical characteristics of obese and lean-matched controls validation and functional studies.

Variable	Obese patients (N =34)	Lean-matched controls (N =34)	P value
Age (years)	38.97 ± SD 12.29	38.05 ± SD 11.44	0.854
Females (%)	82.35%	82.35%	1
BMI***	46.58 ± SD 6.62	22.14 ± SD 1.75	<0.0001
Diabetes (%)	2.94%	0%	-
HbA1c (%)	5.3 ± SD 0.44 (n=29)	-	-
Laboratory Measurements			
Hemoglobin (g/dl)	14.11 ± SD 1.02	13.75 ± SD 1.24 (n=32)	0.167
Leukocytes/µL*	8.65 ± SD 2.22	6.8 ± SD 3.99 (n=32)	0.0133
Platelets/µL	259350 ± SD 60980	242750 ± SD 51570 (n=32)	0.205
Mean Platelet Volume (fL)**	8.47 ± SD 1.16	9.63 ± SD 1.44 (n=31)	0.0022
Glucose (mg/dl)	88.76 ± SD 14.23	83.97 ± SD 8.45 (n=31)	0.111
Creatinin (mg/dl)	0.68 ± SD 0.14	0.74 ± SD 0.12 (n=31)	0.083
Cholesterol (mg/dl)	186.8 ± SD 33.27	197.37 ± SD 34.54 (n=32)	0.197
Chronic Treatments			
Antiplatelets (%)	0%	0%	
ACE Inhibitors (%)	6.25%	0%	
Benzodiazepines (%)	21.8%	0%	
Celecoxib (%)	3.1%	0%	
Statins (%)	3.1%	3.1%	
Antidepressants (%)	6.25%	3.1%	
Muscle relaxants (%)	3.1%	0%	

a. Data are presented as the median ± SD or percentage of patients. * $p<0.05$; ** $p<0.001$; *** $p<0.0001$.

b. Patients with glucose levels below 80 mg/dL were not requested HbA1c measuring.

Supplementary Table 3: Platelet proteins differentially regulated in obese patients versus lean healthy controls.

Uniprot code	Name	Biological process	Spot	Fold Change
**ACTB_HUMAN**	Actin, cytoplasmic 1	Platelet aggregation	4405<br>4325<br>2021<br>2550<br>4461	+1.3<br>+1.3<br>+1.2<br>+1.2<br>+1.2
**ACTG_HUMAN**	Actin, cytoplasmic 2	Platelet aggregation	2550<br>4461	+1.2<br>+1.2
**ACTN1_HUMAN**	Alpha-actinin-1	Platelet degranulation and platelet formation	4465<br>4405<br>4403	+1.2<br>+1.3<br>+1.3
**ALBU_HUMAN**	Serum albumin	Regulation of the colloidal osmotic pressure of blood	4424<br>4356	-1.2<br>+1.3
**ANXA5_HUMAN**	Annexin A5	Hemostasis	4356	+1.3
**ARK72_HUMAN**	Aflatoxin B1 aldehyde reductase member 2	Carbohydrate metabolic process	2898	-1.2
**DCTN2_HUMAN**	Dynactin subunit 2	Cell proliferation	4354	-1.2
**DPYL2_HUMAN**	Dihydropyrimidinase-related protein 2	Endocytosis	1858	-1.2
**FIBB_HUMAN**	Fibrinogen beta chain	Platelet aggregation	2547<br>2077	+1.2<br>+1.2
**FIBG_HUMAN**	Fibrinogen gamma chain	Platelet aggregation	4465<br>4325<br>2232<br>4304<br>4344	+1.2<br>+1.3<br>+1.3<br>+1.2<br>+1.2
**GELS_HUMAN**	Gelsolin	Actin filament capping	4508<br>4505	-1.2<br>-1.2
**GPDM_HUMAN**	Glycerol-3-phosphate dehydrogenase, mitochondrial	Triglyceride catabolic process	4400	-1.4
**HEM2_HUMAN**	Delta-aminolevulinic acid dehydratase	Response to oxidative stress	2932	-1.2
**HSPB1_HUMAN**	Heat shock protein beta-1	Negative regulation of oxidative stress-induced intrinsic apoptotic signaling pathway	4361<br>3287	-1.2<br>-1.2
**ITA2B_HUMAN**	Integrin αIIb	Platelet aggregation	993<br>1006	+1.2<br>+1.2
**LYSC_HUMAN**	Lysozyme C	Inflammatory response	4320	-1.3
**ODO2_HUMAN**	Dihydrolipoyllysine-residue succinyltransferase	Catalytic activity	2077	+1.2

	component of 2-oxoglutarate dehydrogenase complex, mitochondrial			
**PDLI1_HUMAN**	PDZ and LIM domain protein 1	Response to oxidative stress	2782	-1.2
**PDIA6_HUMAN**	Protein disulfide-isomerase A6	Apoptotic cell clearance	4354	-1.2
**PGM2_HUMAN**	Phosphoglucomutase-2	Glucose metabolic process	1711	-1.2
**PI42A_HUMAN**	Phosphatidylinositol 5-phosphate 4-kinase type-2 alpha	Phospholipid metabolic process	4359	-1.2
**PP1R7_HUMAN**	Protein phosphatase 1 regulatory subunit 7	Positive regulation of preotein dephosphorylation	4465	+1.2
**SEPT2_HUMAN**	Septin-2	Regulation of L-glutamate transport	2547	+1.2
**SEP11_HUMAN**	Septin-11	Cell cycle	4359	-1.2
**STIP1_HUMAN**	Stress-induced-phosphoprotein 1	Protein complex	1858	-1.2
**TBB1_HUMAN**	Tubulin beta-1 chain	Spindle assembly	4354	-1.2
**TPM1_HUMAN**	Tropomyosin alpha-1 chain	Actin-binding	4424<br>4348	-1.2<br>-1.2
**TSP1_HUMAN**	Thrombospondin-1	Platelet desgranulation, platelet activation and reactive oxygen species metabolic process	993<br>4405<br>4321<br>4311<br>4403	+1.2<br>+1.3<br>+1.2<br>+1.2<br>+1.3
**TXNL1_HUMAN**	Thioredoxin-like protein 1	Cell redox homeostasis	2867	+1.2
**TYPH_HUMAN**	Thymidine phosphorylase	Angiogenesis	2021	+1.2
**VINC_HUMAN**	Vinculin	Platelet aggregation and platelet degranulation	933<br>1006	+1.2<br>+1.2
**VP37B_HUMAN**	Vacuolar protein sorting-associated protein 37B	Involved in cell growth and differentiation	2932	-1.2
**ZYX_HUMAN**	Zyxin	Regulation of inflammatory response	4491<br>4339	-1.2<br>-1.3

[a.] A positive fold change indicates that the protein feature is up-regulated in obese patients, whereas a negative fold change indicates that the spot is down-regulate.

Supplementary Table 4. Additional data on MS protein identification

Spot	MS method	N†/ % ‡	Mascot Score	Peptides identified by MS		Identified protein	Accession number	MW (exp) / MW(theo)	pI (exp) / pI(theo)	Fold change
				M + H	Sequence					
993	LC-MS	9/10,7	519	561	ALSNVEGFER	Integrin alpha-IIb	ITA2B_HUMAN	141.3/113.3	4.78/5.1	+1.2
				666	FGSAIAPLGDLDR					
				458	AEAQVELR					
				594	DGYNDIAVAAPYGGPSGR					
				518.301	VYLFLQPR					
				611.7923	NVGSQTLQTFK					
				539.564	HDLLVGAPLYMESR					
				751.8823	GQVLVFLGQSEGLR					
				803.3217	TPVGSCFLAQPESGR					
		5/5.9	270.1	530.2687	KDHSGQVFSVVSNGK	Thrombospondin-1	TSP1_HUMAN	141.3/129.3	4.78/4.6	
				808.8687	GGVNDNFQGVLQNVR					
				623.8222	TIVTTLQDSIR					
				625.9483	FTGSQPFGQGVEHATANK					
				604.2803	SITLFVQEDR					
		4/4.3	215.6	553,3259	SLGEISALTSK	Vinculin	VINC_HUMAN	141.3/123.7	4.78/5.4	
				729,3563	AQQVSQGLDVLTAK					
				587,3057	ALASQLQDSLK					
				635,3227	AVAGNISDPGLQK					
1006	LC-MS	3/2.9	120.9	437	GNDIIAAAK	Vinculin	VINC_HUMAN	140.20/123.7	4.81/5.4	+1.2
				635	AVAGNISDPGLQK					
				560	STVEGIQASVK					
		2/1.7	81.0	458	AEAQVELR	Integrin alpha-IIb	ITA2B_HUMAN	140.20/113.3	4.81/5.1	
				561,2335	ALSNVEGFER					
1711	LC-MS	5/7.5	215.1	600	IVLANDPDADR	Phosphoglucomutase-2	PGM2_HUMAN	67.34/32.72	6.08/6.15	-1.2
				528	DLTTGYDDSQPDKK					
				668	DTYMLSSTVSSK					
				728	DLTTGYDDSQPDK					
				438,2078	AAMGPGISR					
1858	LC-MS	4/6.3	161.0	601	ETKPEPMEEDLPENK	Stress-Induces-phosphoprotein	STIP1_HUMAN	62.85/62.6	6.08/6.4	-1.2
				532	EGLQNMEAR					
				503	AAALEFLNR					
				644	KETKPEPMEEDLPENK					
2021	LC-MS	2/7.7	95.1	652	VAPEEHPVLLTEAPLNPK	Actin, Cytoplasmic 1	ACTB_HUMAN	60.17/41.7	5.2/5.2	+1.2

				600	DSYVGDEAQSK					
		1/2.9	52.8	477	VAAALTAMDKPLGR	Tymidine Phosphorylase	TYPH_HUMAN	63.54/49.9	5.2/5.2	
2077	LC-MS	4/12.4	199.2	885<br>513<br>651<br>655	DNENVVNEYSSELEK<br>AHYGGFTVQNEANK<br>EEAPSLRPAPPPISGGGYR<br>QGFGNVATNTDGK	Fibrinogen Beta	FIBB_HUMAN	59.06/55.9	5.5/9.3	+1.2
		1/2.9	62.9	885	AKPAEAPAAAAPK	Dihydrolipoyllysine-Residue Succinyltransferaes	ODO2_HUMAN	59.06/48.7	5.5/9.9	
2232	LC-MS	6/17.2	254.2	345<br>576<br>846<br>498<br>757,3377<br>431,8508	VGPEADKYR<br>TSTADYAMFK<br>AIQLTYNPDESSKPNMIDAATLK<br>YEASILTHDSSIR<br>YLQEIYNSNNQK<br>QSGLYFIKPLK	Fibrinogen Gamma	FIBG_HUMAN	50.54/51.5	5.03/5.3	+1.3
2547	LC-MS	49.8	219.1	513<br>655<br>359<br>618	AHYGGFTVQNEANK<br>QGFGNVATNTDGK<br>ISQLTR<br>TMTIHNGMFFSTYDR	Fibrinogen Beta	FIBB_HUMAN	30.32/55.9	6.06/9.3	+1.2
		3/12.2	146.6	816,6186<br>401,89<br>451,5414	MQAQMQMQMQGGDGDGGALG<br>HHV<br>YLHDESGLNR<br>ILDEIEEHNIK	Septin-2	SEPT2_HUMAN	30.32/41.5	6.06/6.1	
2550	MALDI	9/24	136	795,54<br>976,54<br>1132,63<br>1198,81<br>1791,04<br>1954,23<br>2231,23	IIAPPER<br>AGFAGDDAPR<br>GYSFTTTAER<br>AVFPSIVGRPR<br>SYELPDGQVITIGNER<br>VAPEEHPVLLTEAPLNPK<br>DLYANTVLSGGTTMYPGIADR	Actin, Cytoplasmic 1	ACTB_HUMAN	30.16/42.05	5.26/5.29	+1.2<br><br>OR
		9/24	136	795,54<br>976,54<br>1132,63<br>1198,81<br>1791,04<br>1954,23	IIAPPER<br>AGFAGDDAPR<br>GYSFTTTAER<br>AVFPSIVGRPR<br>SYELPDGQVITIGNER<br>VAPEEHPVLLTEAPLNPK	Actin, Cytoplasmic 2	ACTG_HUMAN	30.16/41.79	5.26/5.31	

				2231,23	DLYANTVLSGGTTMYPGIADR					
2787	LC-MS	3/10.9	201.0	800<br>480<br>588	SAMPFTASPASSTTAR<br>VAASIGNAQK<br>DFEQPLAISR	PDZ and LIM domain protein 1	PDLI1_HUMAN	13.71/36.07	6.18/6.56	-1.2
2867	LC-MS	6/28	278.7	637<br>573<br>565<br>681<br>584,7476<br>733,6486	QHLENDPGSNEDTDIPK<br>FQGPDNGQGPK<br>SMDFEEAER<br>GYMDLMPFINK<br>IAPAFSSMSNK<br>VGVKPVGSDPDFQPELSGAGSR	Thioredoxin-like	TXNL1_HUMA N	13.60/32.2	4.92/4.7	+1.2
2898		5/11.4	265.4	301<br>355<br>411<br>642<br>538,2157	SLKPDSVR<br>SQLETSLKR<br>RMDAPASAAAVR<br>VASVLGTMEMGR<br>MDAPASAAAVR	Aflatoxin B1 aldehyde reductase member 2	ARK72_HUMAN	14.21/39.58	6.37/6.70	-1.2
2932	LC-MS	2/7.7	122.5	451<br>663	MEETQNVQLNK<br>IEEDTENMAEK	Vacuolar Protein	VP37B_HUMAN	15.39/31.3	6.21/7.6	-1.2
		2/6.4	86.8	525<br>598	LAEVALAYAK<br>AAVLEAMTAFR	Delta-aminolevulinc	HEM2_HUMAN	15.39/36.3	6.21/6.4	
3287	MALDI	12/42	383	831,58<br>987,68<br>1075,66<br>1104,6<br>1163,72<br>1643,95<br>1784,06<br>1906,15	VPFSLLR<br>RVPFSLLR<br>QLSSGVSEIR<br>QDEHGYISR<br>LFDQAFGLPR<br>AQLGGPEAAKSDETAAK<br>VSLDVNHFAPDELTVK<br>LATQSNEITIPVTFESR	Heat shock protein beta-1	HSPB1_HUMAN	24.74/22.86	5.89/5.98	-1.2
4304	MALDI	12/26	98	851,5<br>1034,61<br>1037,63<br>1045,66<br>1117,62<br>1150,61<br>1194,59<br>1293,86<br>1513,85	NWIQYK<br>VGPEADKYR<br>KMLEEIMK<br>TSEVKQLIK<br>VELEDWNGR<br>TSTADYAMFK<br>DNCCILDER<br>QSGLYFIKPLK<br>YLQEIYNSNNQK	Fibrinogen Gamma	FIBG_HUMAN	49.13/52.101	4.87/ 5.37	+1.2

				1683,1<br>2536,46	IHLISTQSAIPYALR<br>AIQLTYNPDESSKPNMIDAATLK					
4311	LC-MS	1/0.9	45.5	624	TIVTTLQDSIR	Thrombospondin-1	TSP1_HUMAN	129.61/129.3	4.95/4.6	+1.2
4320	MALDI	6/26	72	788,44<br>1012,49<br>1179,64<br>1400,72	AWVAWR<br>WESGYNTR<br>VFERCELAR<br>STDYGIFQINSR	Lysozyme C	LYSC_HUMAN	77.72/16.98	4.35/9.38	-1.3
4321	LC-MS	4/4	200.7	530<br>516<br>373<br>809	KDHSGQVFSVVSNGK<br>GPDPSSPAFR<br>DLASIAR<br>GGVNDNFQGVLQNVR	Thrombospondin-1	TSP1_HUMAN	127.83/129.3	4.99/4.6	+1.2
4325	LC-MS	7/25.3	374.6	896<br>506<br>589<br>323<br>566,7203<br>652,0153<br>744,3243	SYELPDGQVITIGNER<br>QEYDESGPSIVHR<br>EITALAPSTMK<br>LDLAGR<br>GYSFTTTAER<br>VAPEEHPVLLTEAPLNPK<br>DLYANTVLSGGTTMYPGIADR	Actin, Cytoplasmic 1	ACTB_HUMAN	20.71/41.7	5.49/5.2	+1.3
		2/5.3	82.8	497,8928<br>431,9183	YEASILTHDSSIR<br>QSGLYFIKPLK	Fibrinogen Gamma	FIBG_HUMAN	20.71/51.5	5.49/5.3	
4339	MALDI	12/20	214	749,46<br>775,49<br>881,5<br>1037,67<br>1039,71<br>1515,84<br>1710,03<br>1989,16<br>2597,49	FTPVASK<br>FSPVTPK<br>CHQPLAR<br>SPGAPGPLTLK<br>FGPVVAPKPK<br>FSPGAPGGSGSQPNQK<br>QHPVPPPAQNQNQVR<br>VNPFRPGDSEPPPAPGAQR<br>LGHPEALSAGTGSPQPPSFTYAQQ<br>R	Zyxin	ZYX_HUMAN	89.89/62.43	6.21/8.59	-1.3
4344	MALDI	6/15	62	1045,71<br>1293,92<br>1491,96<br>1513,94<br>2536,6	TSEVKQLIK<br>QSGLYFIKPLK<br>YEASILTHDSSIR<br>YLQEIYNSNNQK<br>AIQLTYNPDESSKPNMIDAATLK	Fibrinogen gamma	FIBG_HUMAN	22.18/52.101	5.45/5.37	+1.2

4348	MALDI	17/34	166	722,41<br>744,51<br>777,41<br>810,46<br>875,53<br>894,55<br>926,52<br>1073,66<br>1109,59<br>1186,77<br>1243,76<br>1314,87<br>1475,88<br>1728,04	AEFAER<br>LATALQK<br>AADESER<br>KMQMLK<br>SLEAQAEK<br>YEEVARK<br>HIAEDADR<br>LDKENALDR<br>AADESERGMK<br>LVIIESDLER<br>IQLVEEELDR<br>KLVIIESDLER<br>MEIQEIQLKEAK<br>IQLVEEELDRAQER	Tropomyosin alpha-1 chain	TPM1_HUMAN	13.10/32.74	4.71/4.69	-1.2
4354	LC-MS	7/20.4	351.8	708<br>473<br>848<br>668<br>779,9624<br>544,6017<br>580,3036	EVDQQLLSVQTR<br>GASALQLER<br>ALSVAELTQQMFDAR<br>ISVYYNEAYGR<br>AVLEEDEEVTEEAEMEPEDK<br>AVLVDLEPGTMDSIR<br>LAVNMVPFPR	Tubulin beta-1	TBB1_HUMAN	57.70/50.3	5.01/4.9	+1.2
		4/10.5	163.6	384,8861<br>405,163<br>516,2628<br>883,4082	QQLVASHLEK<br>GLDFSDR<br>LTELETAVR<br>ENLATVEGNFASIDER	Dynactin Subunit2	DCTN2_HUMAN	57.70/44.2	5.01/5	
		3/8.6	145.7	397,8601<br>808,3937<br>576,1825	NRPEDYQGGR<br>GSTAPVGGGAFPTIVER<br>GESPVDYDGGR	Protein Disulfide-isomerase	PDIA6_HUMAN	557.70/48.1	5.01/4.8	
4356	MALDI	22/54	275	744,44<br>775,46<br>826,54<br>893,54<br>903,56<br>954,61<br>1001,68<br>1014,59	TPEELR<br>ADAETLR<br>SVSHLRK<br>QEISAAFK<br>ADAETLRK<br>FITIFGTR<br>VLTEIIASR<br>LYDAYELK	Annexin A5	ANXA5_HUMAN	18.05/35.97	4.85/4.93	+1.3

				1106,67<br>1143,78<br>1155,67<br>1172,76<br>1234,77<br>1290,73<br>1340,71<br>1705,02<br>1750<br>2888,45	SEIDLFNIR<br>LIVALMKPSR<br>GAGTDDHTLIR<br>MLVVLLQANR<br>SEIDLFNIRK<br>NFATSLYSMIK<br>GTVTDFPGFDER<br>GLGTDEESILTLLTSR<br>SIPAYLAETLYYAMK<br>QVYEEEYGSSLEDDVVGDTSGYY QR					
		6/9	121	927,57<br>1149,69<br>1640,06<br>1911,03	YLYEIAR<br>LVNEVTEFAK<br>KVPQVSTPTLVEVSR<br>RPCFSALEVDETYVPK	Serum Albumin	ALBU_HUMAN	18.05/69.3	4.85/5.9	
4359	LC-MS	6/15.4	340.2	653<br>696<br>690<br>644<br>519,2193<br>612,7728	AAAQLLQSQAQQSGAQQTK<br>KAAAQLLQSQAQQSGAQQTK<br>ELEEEVNNFQK<br>STLMDTLFNTK<br>FESDPATHNEPGVR<br>SYELQESNVR	Septin-11	SEP11_HUMAN	54.15/49.39	6.62/6.36	-1.2
		2/5.2	88.7	590,2191<br>578,24	DNDFINEGQK<br>SAPLPNDSQAR	Phosphatidylinositol 5-phosphate 4-kinase type-2 alpha	PI42A_HUMAN	54.15/46.22	6.62/6.50	
4361	MALDI	12/42	383	831,58<br>987,68<br>1075,66<br>1104,6<br>1163,72<br>1643,95<br>1784,06<br>1906,15	VPFSLLR<br>RVPFSLLR<br>QLSSGVSEIR<br>QDEHGYISR<br>LFDQAFGLPR<br>AQLGGPEAAKSDETAAK<br>VSLDVNHFAPDELTVK<br>VSLDVNHFAPDELTVK<br>LATQSNEITIPVTFESR	Heat shock protein beta-1	HSPB1_HUMAN	24.29/22.86	5.89/5.98	-1.2
4400	LC-MS	9/10.9	484.7	392<br>581<br>633	SSYVLSK<br>MNLAIALTAAR<br>SMAEDTINAAVK	Glycerol-3-phosphate dehydrogenase, mitochondrial	GPDM_HUMAN	64.70/80.82	6.37/7.58	-1.4

				488<br>417,2137<br>474,2226<br>574,3066<br>332,6644<br>529,6788	QEQLETAR<br>IVELMGR<br>TAEENLDR<br>GFITIVDVQR<br>AFEVAK<br>DDFSSGTSSR					
4403	LC-MS	7/7.3	129.30	826<br>626<br>667<br>516<br>422,7228<br>531,7262<br>530,2393	DCVGDVTENQICNK<br>FTGSQPFGQGVEHATANK<br>LCNNPTPQFGGK<br>GPDPSSPAFR<br>ELANELR<br>FQDLVDAVR<br>KDHSGQVFSVVSNGK	Thrombospondin-1	TSP1_HUMAN	124.87/129.3	5.08/4.6	+1.3
		2/2.7	103	493,1916<br>677,7483	LSNRPAFMPSEGR<br>GISQEQMNEFR	Alpha-actinin-1	ACTN1_HUMA<br>N	124.87/103	5.08/5.1	
4405	LC-MS	7/7.8	414.7	624<br>809<br>667<br>516<br>625,9361<br>604,2892<br>530,2655	TIVTTLQDSIR<br>GGVNDNFQGVLQNVR<br>LCNNPTPQFGGK<br>GPDPSSPAFR<br>FTGSQPFGQGVEHATANK<br>SITLFVQEDR<br>KDHSGQVFSVVSNGK	Thrombospondin-1	TSP1_HUMAN	126.35/129.3	5.04/4.6	+1.3
		2/7.5	74.5	652,0124<br>488,7376	VAPEEHPVLLTEAPLNPK<br>AGFAGDDAPR	Actin, Cytoplasmic 1	ACT<br>B_HUMAN	126.35/41.7	5.04/5.2	
		1/1.6	55.2	769,3351	FAIQDISVEETSAK	Alpha-actinin-1	ACTN1_HUMA<br>N	126.35/103	5.04/5.1	
4424	LC-MS	8/20.8	403.6	574<br>373<br>825<br>404<br>358,5302<br>622,3107<br>657,8473<br>593,8076	MEIQEIQLK<br>LATALQK<br>DEEKMEIQEIQLK<br>EDRYEEEIK<br>LDKENALDR<br>IQLVEEELDR<br>KLVIIESDLER<br>LVIIESDLER	Trompomyosin Alpha-1	TPM1_HUMAN	22.02/32.7	4.71/4.5	-1.2
		1/ 2.5	73.4	547,3077	KVPQVSTPTLVEVSR	Serum Albumin	ALBU_HUMAN	22.02/69.3	4.71/5.9	
4461	MALDI	15/32	416	795,53	IIAPPER	Actin, Cytoplasmic 1	ACTB_HUMAN	20.99/42.05	5.29/5.29	+1.2

				923,63<br>1014,57<br>1132,61<br>1177,69<br>1516,82<br>1791,03<br>1911,01<br>1954,21<br>2231,23	IIAPPERK<br>DLTDYLMK<br>GYSFTTTAER<br>EITALAPSTMK<br>QEYDESGPSIVHR<br>SYELPDGQVITIGNER<br>MDDDIAALVVDNGSGMCK<br>VAPEEHPVLLTEAPLNPK<br>DLYANTVLSGGTTMYPGIADR					OR
				795,53<br>923,63<br>1014,57<br>1132,61<br>1177,69<br>1516,82<br>1791,03<br>1851,98<br>1954,21<br>2231,23	IIAPPER<br>IIAPPERK<br>DLTDYLMK<br>GYSFTTTAER<br>EITALAPSTMK<br>QEYDESGPSIVHR<br>SYELPDGQVITIGNER<br>EEEIAALVIDNGSGMCK<br>VAPEEHPVLLTEAPLNPK<br>DLYANTVLSGGTTMYPGIADR	Actin, Cytoplasmic 2	ACTG_HUMAN	20.99/42.05	5.29/5.31	
4465	LC-MS	6/13.5	300.7	441<br>757<br>346<br>432<br>351,8449<br>497,9094	LDGSVDFK<br>YLQEIYNSNNQK<br>VGPEADKYR<br>QSGLYFIKPLK<br>KMLEEIMK<br>YEASILTHDSSIR	Fibrinogen Gamma	FIBG_HUMAN	49.29/51.5	4.81/5.3	+1.2
		2/6.7	124.1	555,2467<br>575,8878	SLETVYLER<br>GAGQQQSQEMMEVDR	Protein Phosphatase 1	PP1R7_HUMAN	49.29/41.5	4.81/4.7	
		2/5.1	100.8	452,1944<br>442,5648	GISQEQMNEFR<br>RDQALTEEHAR	Alpha-actinin-1	ACTN1_HUMA<br>N	49.29/103	4.81/5.1	
4491	LC-MS	4/10.8	213.0	571<br>539<br>664<br>506	QHPVPPPAQNQNQVR<br>GPPASSPAPAPK<br>VNPFRPGDSEPPPAPGAQR<br>FSPGAPGGSGSQPNQK	Zyxin	ZYX_HUMAN	91.07/61.2	6.13/6.2	-1.2
4505	MALDI	23/20	262	747,44<br>839,46<br>839,46	ANSAGATR<br>MDAHPPR<br>LFACSNK	Gelsolin	GELS_HUMAN	102.33/86.04	5.5/5.9	-1.3

				847,47<br>850,54<br>859,51<br>887,57<br>913,66<br>967,56<br>998,62<br>1074,62<br>1078,61<br>1165,69<br>1254,8<br>1275,83<br>1319,81<br>1349,76<br>1830,11<br>2479,41	LFQVKGR<br>KGGVASGFK<br>GIRDNER<br>TGAQELLR<br>RTPITVVK<br>KMDAHPPR<br>EPGLQIWR<br>EGGQTAPASTR<br>YIETDPANR<br>LFACSNKIGR<br>AGKEPGLQIWR<br>HVVPNEVVVQR<br>AGALNSNDAFVLK<br>YIETDPANRDR<br>QTQVSVLPEGGETPLFK<br>VSNGAGTMSVSLVADENPFAQG ALK					
4508	LC-MS	8/11.1	85.6	379<br>444<br>556<br>426<br>610,6398<br>660,3282<br>378,2259<br>539,7373	TPITVVK<br>TGAQELLR<br>DSQEEEKTEALTSAK<br>HVVPNEVVVQR<br>QTQVSVLPEGGETPLFK<br>AGALNSNDAFVLK<br>AVEVLPK.A<br>R.YIETDPANR	Gelsolin	GELS_HUMAN	102.93/85.6	5.5/5.9	-1.2

a. Identifications were by MALDI-MS/MS in 11 spots and by LC-MS/MS in 24 spots. † Number of matched peptides. ‡ % coverage of full length protein by tryptic peptides. All differential proteins have a p value lower than 0.05.

Supplementary Table 5. Aggregation data corresponding to Figure 3, representing agonists, condition, doses, cohort and p value (Mann-Whitney test).

	Condition	Doses (µg/mL)	n	p-value
CRP	PRP	0.1<br>0.15<br>0.2	18<br>20<br>18	0.049<br>0.0048<br>0.0083
CRP	Washed platelets	0.4<br>0.5<br>1	20<br>20<br>20	0.356<br>0.092<br>0.0015
COLL	PRP	0.5<br>0.75<br>1	20<br>20<br>17	0.059<br>0.0002<br>0.022
COLL	Washed platelets	1<br>2<br>3	20<br>19<br>19	0.56<br>0.81<br>0.60

a. CRP: collagen-related peptide; COLL: collagen.

Supplementary Table 6. Aggregation data corresponding to Supplementary Figure 2, representing agonists, condition, doses, cohort and p value (Mann-Whitney test).

	Condition	Doses	n	p-value
RHO	PRP	25 nM<br>50 nM	20<br>20	0.61<br>0.08
RHO	Washed platelets	25 nM<br>50 nM<br>100 nM	20<br>20<br>18	0.71<br>0.91<br>0.35
ADP	PRP	2 µM<br>3 µM	11<br>8	0.7<br>0.1
AA	PRP	0.3 mM<br>0.5 mM	9<br>5	0.15<br>0.66
THR	Washed platelets	0.5 u/mL<br>0.75 u/mL	14<br>20	0.8<br>0.3

a. RHO: rhodocytin; AA: arachidonic acid; THR: thrombin.

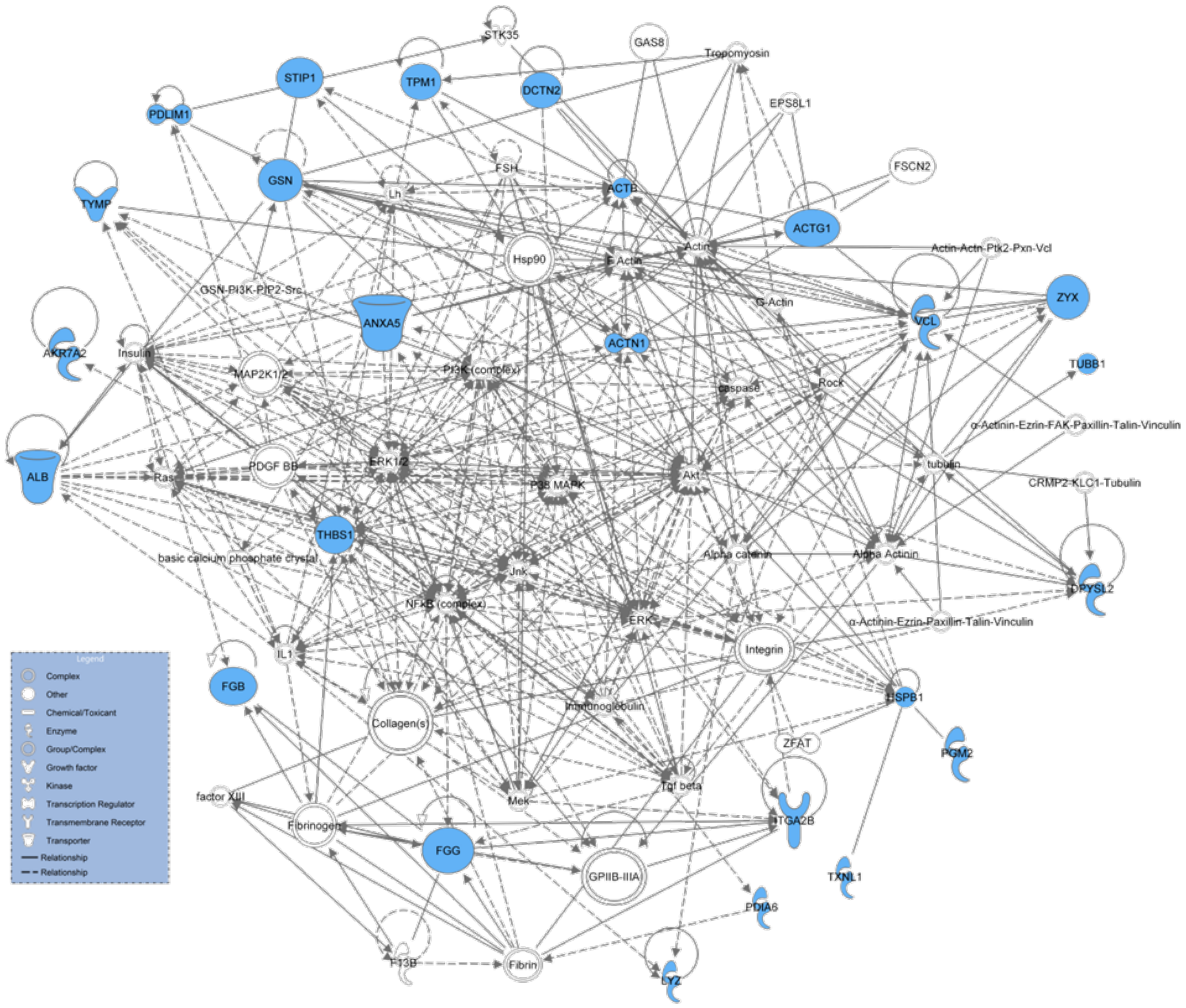

Supplementary Fig. 1: Ingenuity Pathway Analysis (IPA) of the proteomic results identifies a protein network related to Cell-To-Cell Signaling and Interaction, Hematological System Development and Function and Inflammatory Response altered in obesity. Proteins identified by differential analysis are shown as shaded nodes with their gene names. Solid lines represent direct interactions, dotted represent indirect interactions.

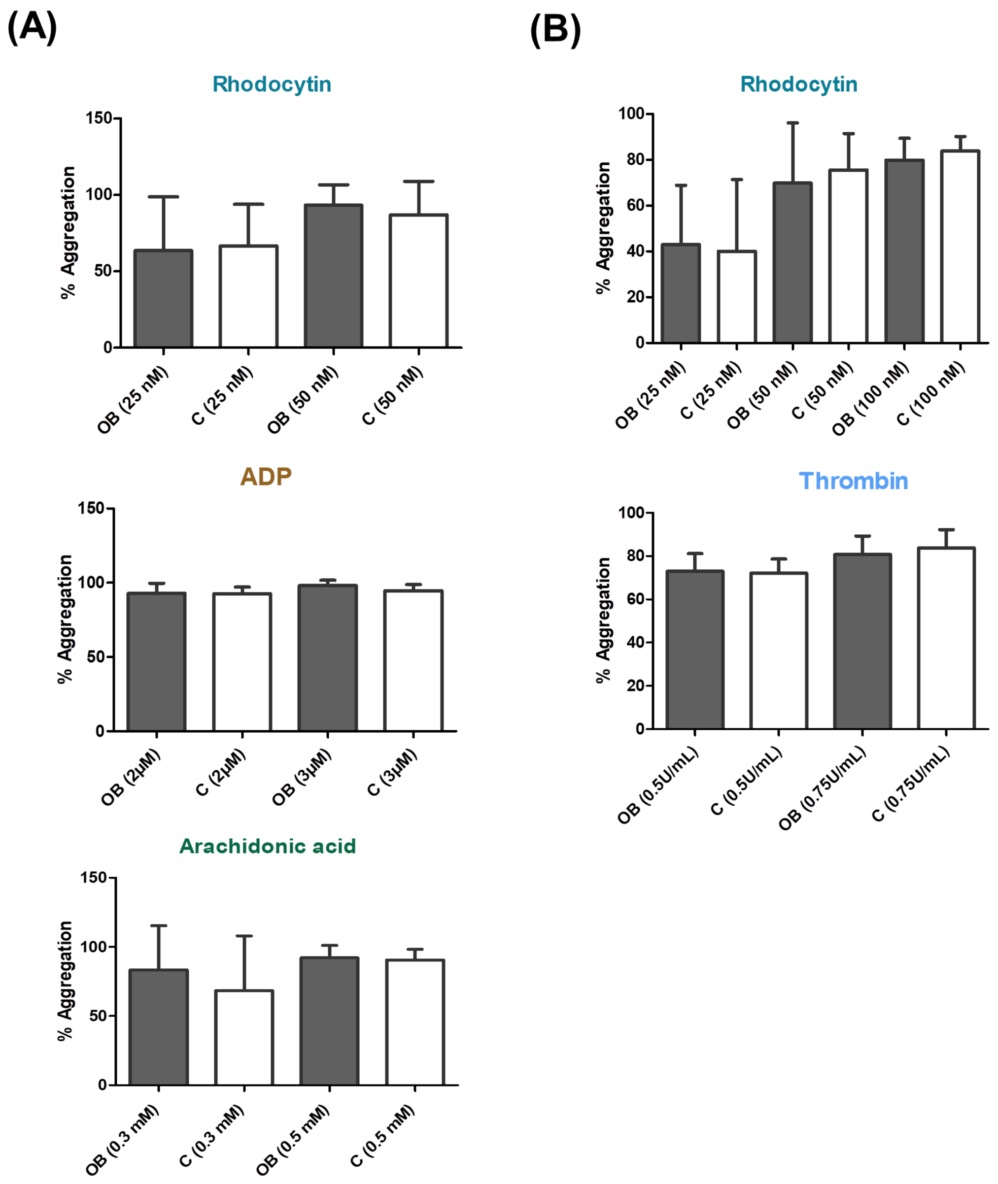

Supplementary Fig. 2: Aggregation studies showing no differences between platelets from obese and lean individuals in response to various agonists. PRP and washed platelets were stimulated with rhodocytin (CLEC-2 agonist); or with non-SFKs-mediated signaling pathways agonists such as ADP, arachidonic acid and thrombin (A) PRP was stimulated with rhodocytin (25nM and 50nM), ADP (2 and 3 µM) and arachidonic acid (0.3 and 0.5 mM) to trigger aggregation. (B) Washed platelets were stimulated with rhodocytin (25, 50 and 100nM) and thrombin (0.5 and 0.75 u/mL).

Results are presented as mean ±SD; P-values and cohort data are shown in Supplementary Table 6. OB: obese; C: control.

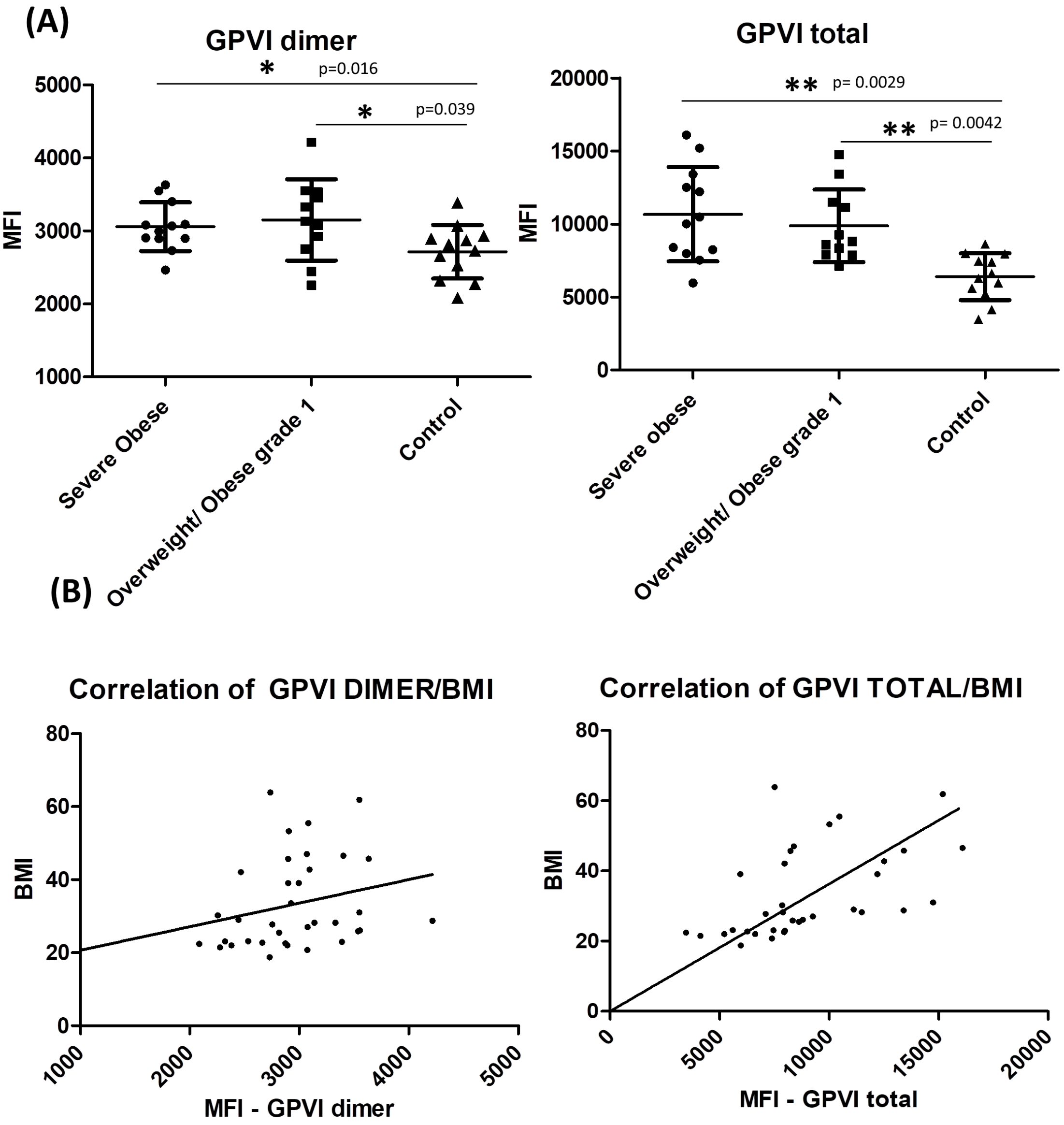

Number of XY Pairs	35
Spearman r	0.3781
95% confidence interval	0.04100 to 0.6379
P value (two-tailed)	0.0251

Number of XY Pairs	35
Spearman r	0.5982
95% confidence interval	0.3217 to 0.7807
P value (two-tailed)	0.0001

Supplementary Fig. 3: Higher expression levels of GPVI (total and dimer) in the surface of platelets from obese and overweight individuals compared to lean controls, with a positive correlation with BMI. The study was performed with a

subgroup of 12 severe obese patients, their 12 lean matched-controls, and an additional group of 11 overweight and obese grade 1 individuals (BMI between 26 and 35). (A) GPVI dimer and total levels of GPVI are increased in the obese groups. Fluorescence values are shown as mean ± SD. Measures were done in triplicate. Significant differences are indicated as follow: *$p<0.05$, **$p<0.01$. (B) Positive correlations between BMI and total GPVI and GPVI dimer were found using Spearman's test.
